# Potent antitumor activity of the novel HSP90 inhibitors AUY922 and HSP990 in neuroendocrine carcinoid cells

**DOI:** 10.3892/ijo.2013.2130

**Published:** 2013-10-04

**Authors:** KATHRIN ZITZMANN, GALINA AILER, GEORGE VLOTIDES, GERALD SPOETTL, JULIAN MAURER, BURKHARD GÖKE, FELIX BEUSCHLEIN, CHRISTOPH J. AUERNHAMMER

**Affiliations:** 1 Department of Internal Medicine II Campus Grosshadern, University-Hospital, Ludwig-Maximilians-University of Munich, D-81377 Munich, Germany; 2 Department of Internal Medicine IV Campus Innenstadt, University-Hospital, Ludwig-Maximilians-University of Munich, D-81377 Munich, Germany

**Keywords:** heat shock protein 90, heat shock protein 90 inhibitors, gastroenteropancreatic, neuroendocrine tumor

## Abstract

The heat shock protein (HSP) 90 chaperone machine involved in numerous oncogenic signaling pathways is over-expressed in cancer cells and is currently being evaluated for anticancer therapy. Neuroendocrine tumors (NETs) of the gastroenteropancreatic (GEP) system comprise a heterogeneous group of tumors with increasing incidence and poor prognosis. Here, we report the antiproliferative effects of the HSP90 inhibitors AUY922 and HSP990 in neuroendocrine tumor cells. Treatment of human pancreatic BON1, bronchopulmonary NCI-H727 and midgut carcinoid GOT1 neuroendocrine tumor cells with increasing concentrations of AUY922 and HSP990 dose-dependently suppressed cell viability. Significant effects on neuroendocrine cell viability were observed with inhibitor concentrations as low as 5 nM. Inhibition of cell viability was associated with the induction of apoptosis as demonstrated by an increase in sub-G1 events and PARP cleavage. HSP90 inhibition was associated with decreased neuroendocrine ErbB and IGF-I receptor expression, decreased Erk and Akt phosphorylation and the induction of HSP70 expression. These findings provide evidence that targeted inhibition of upregulated HSP90 activity could be useful for the treatment of aggressive neuroendocrine tumors resistant to conventional therapy.

## Introduction

Heat shock protein 90 (HSP90) is a molecular chaperone, which in complex with co-chaperones (such as HSP70) forms the HSP90 chaperone machine. This complex has been demonstrated to play an important role in stabilization and activation of numerous key oncogenic client proteins including Akt, MEK, EGFR, ErbB2 and IGF-IR (1,2). The HSP90 monomer consists of 3 domains: the amino terminal domain, which contains the ATP-binding site, the middle domain with docking sites for client proteins and the carboxy terminal region, which resembles a dimerization motif (2). Co-chaperone binding sites are present in all 3 domains whereas drug-binding regions are present in the amino and carboxy terminal domains (2). Normal chaperone function requires dimerization of two HSP90 monomers and recruitment of co-chaperones which regulate the conformational dynamics and activity of the chaperone (2). HSP90 is over-expressed in cancer cells and associated with decreased survival in breast cancer, gastrointestinal stromal tumors and non-small cell lung cancer (3). Given the potential to block multiple oncogenic signaling pathways simultaneously and thus possibly counteract escape mechanisms and resistance to targeted monotherapies, several HSP90 inhibitors are currently undergoing clinical trials in multiple indications as single agents or in combination therapy (2–4).

Neuroendocrine tumors (NETs) of the gastroenteropancreatic (GEP) system comprise a rare group of tumors which accounts for ∼2% of all gastrointestinal tumors (5,6). Over the last decades the incidence of GEP NETs has increased considerably (6,7). Around 25% of all NETs present with distant metastasis at the time of first diagnosis and despite advances in surgical and medical therapy the overall 5-year survival rate remains rather low (∼60%) (6,8,9). Thus, novel therapeutic tools are needed for this heterogeneous group of tumors. Due to high activity of Akt and Erk signaling in NETs and compensatory activation of Akt in response to mTOR and Raf inhibitors, targeting HSP90 could provide a tool to simultaneously suppress both survival pathways (10,11). Furthermore, HSP90 overexpression has recently been reported in NETs and the HSP90 inhibitors 17-AAG and IPI-504 have demonstrated antiproliferative efficacy in several NET cell lines *in vitro* (12,13).

Here we report antiproliferative effects of the novel small molecule HSP90 inhibitors AUY922 and HSP990 and characterize HSP90 downstream signaling in neuroendocrine tumor cells of pancreatic, midgut and bronchopulmonary origin.

## Materials and methods

### Materials

DMEM/F12 media, penicillin and streptomycin were purchased from Gibco/Invitrogen (Karlsruhe, Germany) and RPMI medium was from PAA Laboratories (Pasching, Austria). Fetal bovine serum (FBS) and amphotericin B were from Biochrom (Berlin, Germany), and AUY922 and HSP990 were kindly provided from Novartis Pharma (Basel, Switzerland).

### Cell cultures

All human neuroendocrine cell lines were received and cultured as described (14). Briefly, pancreatic neuroendocrine BON1 tumor cells (kindly provided by R. Göke, Marburg) were cultured in DMEM/F12 (1:1) medium supplemented with 10% FBS, 1% penicillin/streptomycin and 0.4% amphotericin B. Human midgut carcinoid GOT1 cells (kindly provided by Professor Ola Nilsson, Sahlgrenska University Hospital, Gothenburg, Sweden) and human broncho-pulmonary neuroendocrine NCI-H727 tumor cells (purchased from ATCC, Manassas, VA, USA) were both cultured in RPMI medium supplemented with 10% FBS, 1% penicillin/streptomycin and 0.4% amphotericin B. Additional supplements in GOT1 culture medium were 0.135 IU/ml insulin and 5 mg/dl apo-transferrin.

### Assessment of cell viability

Cell viability was assessed as described (14). Briefly, cells were seeded into 96-well plates at densities of 3,000 (BON1), 50,000 (GOT1) and 4,000 (NCIH727) cells per well, respectively, and grown for 24 h. The next day, medium was replaced by serum rich medium (10% FBS) containing various concentrations of AUY922 and HSP990 (0.1, 0.5, 1, 5, 10, 50, 100 nM) and the cells were further incubated for indicated time intervals. Cell viability expressed by metabolic activity was measured with Cell Titer 96 aqueous One Solution Cell Proliferation assay (Promega, Madison, WI, USA) according to the manufacturer’s instructions. Following 3 h of incubation with Cell Titer 96 solution, absorbance at 492 nm was determined using an ELISA plate reader.

### SYBR-DNA-labeling assay

The SYBR-DNA-labeling experiment was performed identically to that described for the Cell Titer 96 aqueous One Solution Cell Proliferation assay. Assays were stopped after indicated time intervals by flicking off the medium and freezing the plate. Cells were stained with 200 *μ*l/well of SYBR-Green^®^ I (Lonza, Basel, Switzerland) 1:4,000 in *aqua destillata* for 30 min in the dark and then quantified by flourimetry at 530 nm with 485 nm excitation, measured using a CytoFluor^®^ Multi-Well Plate Reader Series 4000 (PerSeptive Biosystems, Framingham, MA, USA).

### Cell cycle analysis

Apoptosis and cell cycle distribution were analyzed using flow cytometry as described (14). Briefly, cells were scraped with a rubber policeman, washed with PBS and incubated in staining buffer containing 0.1% sodium citrate, 0.1% Triton X-100 (Sigma) and 50 *μ*g/ml propidium iodide overnight. Sub-G1 events and cell cycle distribution were measured in a fluorescence-activated cell sorter (FACSCalibur, Becton-Dickinson, Franklin Lakes, NJ, USA). Nuclei to the left of the G1-peak containing hypodiploid DNA were considered apoptotic.

### Caspase assay

Activity of effector caspases 3 and 7 was measured with Caspase-Glo 3/7 assay (Promega) according to the manufacturer’s instructions. Following 1 h of incubation with Caspase-Glo 3/7 reagent, luminescence was determined using a plate-reading luminometer.

### Protein extraction and western blot analysis

Protein extraction and western blot analysis were performed as described (14). Briefly, cells were lysed in 500 *μ*l lysis buffer. The lysates were centrifuged for 10 min at 4°C and 13,000 × g and supernatans were adjusted to equal protein loads and diluted 1:1 with SDS sample buffer. Samples were boiled for 5 min and separated on an SDS polyacrylamide gel. Proteins were electrotransferred for 60 min onto PVDF membranes (Immobilone; Millipore, Eschborn, Germany) using a semi-dry western blot technique. After blocking in 2% non-fat dried milk, the membranes were incubated overnight in appropriate dilutions of antibodies against pAkt (Ser 473) (1:20,000), Akt (1:5,000), pErk 1/2 (1:10,000), Erk 1/2 (1:20,000), PARP (1:1,000), IGFR (1:5,000), p70S6K (1:1,000), pp70S6K (1:2,000), 4EBP1 (1:2,000) p4EBP1 (1:1,000) (all from Cell Signaling, Danvers, MA, USA), HSP70 (1:10,000) (Biomol Stressgen, Hamburg, Germany), HSP90 (1:5,000), EGFR (1:1,000), ErbB2 (1:500), ErbB3 (1:1,000) and STAT3 (1:10,000) (all from Santa Cruz, Heidelberg, Germany). After washing with PBS, the membranes were incubated with peroxidise-conjugated secondary antibody (1:25,000) for 2 h. The blots were washed and immersed in the chemiluminescent substrate SuperSignal West Dura (Thermo Scientific, Rockford, IL, USA) and exposed to Super RX Fujifilm (Fujifilm Corporation, Tokyo, Japan).

### Statistical analysis

IC_50_ inhibition values were determined with the use of Prism 6 for May OS X software (www.graphpad.com). Cell cycle phases were analyzed by Cell Quest Software (Becton-Dickinson) and comparisons evaluated using 2-tailed Student’s t-test. Results are expressed as mean ± SD of independently performed experiments. Statistical significance was set at p<0.05.

## Results

### HSP90 expression and inhibitor specificity

**Figure 1.**
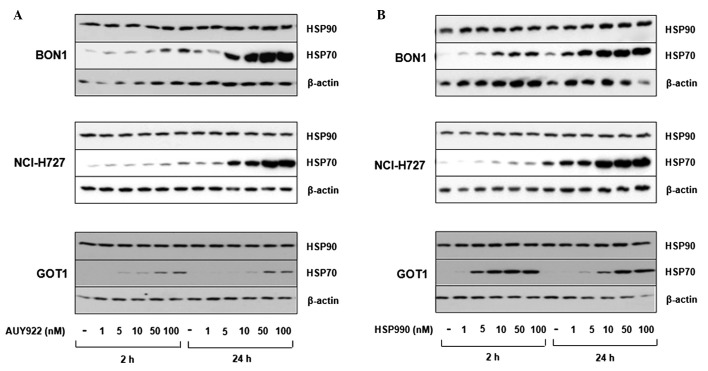
Induction of HSP70 expression by HSP90 inhibition in neuroendocrine tumor cells. BON1, NCI-H727 and GOT1 cells were treated with increasing concentrations (1–100 nM) of the HSP90 inhibitors (A) AUY922 and (B) HSP990 for 2 and 24 h. Subsequently the expression of HSP90 and HSP70 was evaluated by western blot analysis. A representative blot of 3 independently performed experiments is shown.

Western blot analysis revealed BON1, NCI-H727 and GOT1 cells to express easily detectable levels of HSP90, while HSP70 was poorly expressed under baseline conditions. As increased HSP70 expression is a hallmark of specific HSP90 inhibition, we analyzed HSP70 expression after treatment with the HSP90 inhibitors AUY922 and HSP990. Treatment with increasing concentrations (10–100 nM) of both inhibitors induced HSP70 expression in a dose-dependent manner (Fig. 1).

### Inhibition of neuroendocrine cell viability by HSP90 inhibitors

**Figure 2.**
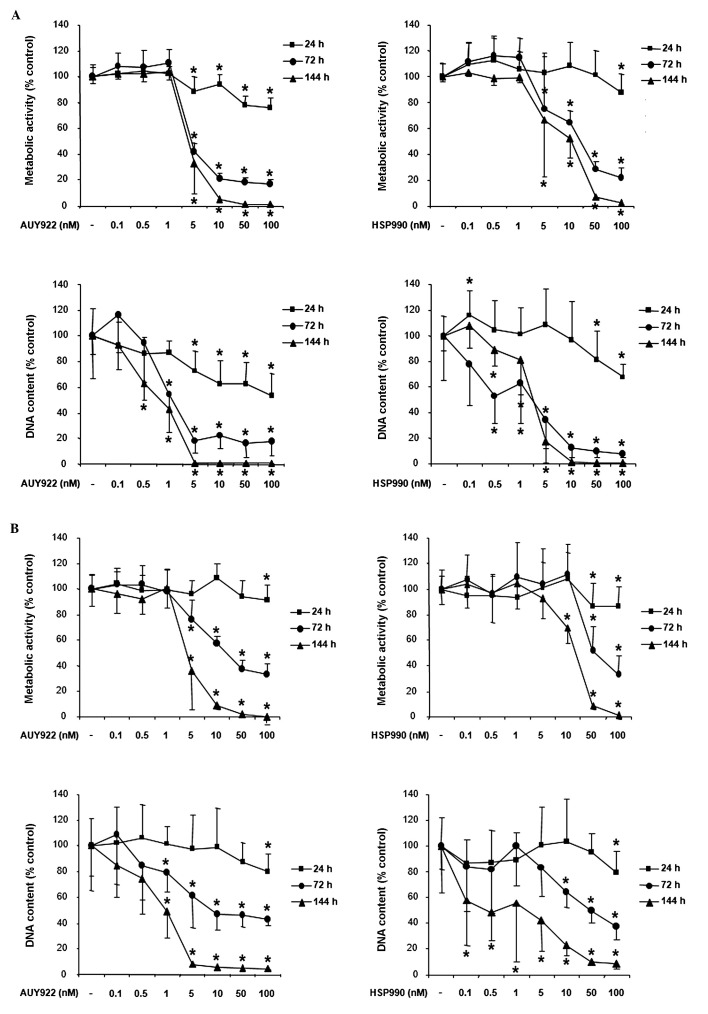

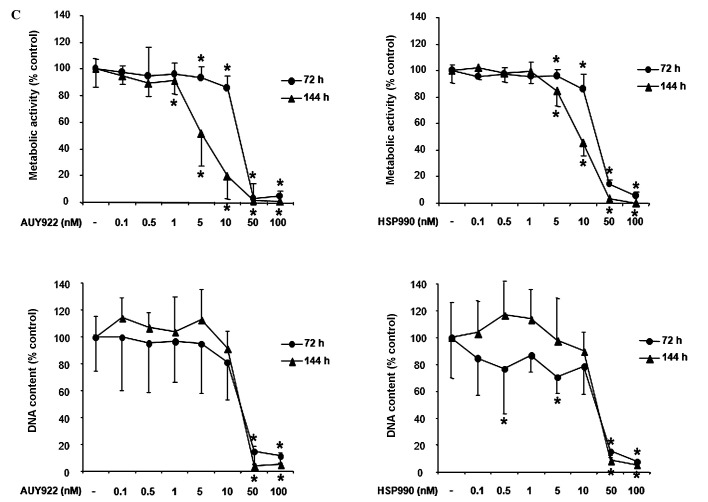
Time- and dose-dependent effects of HSP90 inhibition on neuroendocrine cell viability. (A) BON1, (B) NCI-H727 and (C) GOT1 cells were treated with increasing concentrations (0.1–100 nM) of the HSP90 inhibitor AUY922 or HSP990 for indicated time points (24–144 h). Cell viability based on metabolic activity was measured with Cell Titer 96 kit (Promega; upper panels). Cell proliferation based on DNA content was determined by fluorescence SYBR-Green DNA labelling (lower panels). The mean values ± SD of 3 independently performed experiments in sextuplicates (n=18) are demonstrated. ^*^p<0.05 versus untreated control.

Treatment of human pancreatic neuroendocrine BON1 tumor cells with the HSP90 inhibitor AUY922 dose-dependently suppressed cell viability as assessed by measurement of metabolic activity and DNA content (Fig. 2A, left panel). Significant effects were observed at all time points tested (24, 72 and 144 h) beginning at AUY922 concentrations as low as 5 nM (suppression of metabolic activity to ∼89, 42 and 36% compared to non-treated controls, respectively; p<0.05) and peaked at the highest dose tested (100 nM; suppression of metabolic activity to ∼75, 17 and 1%, respectively; p<0.05). Similar to AUY922, the HSP90 inhibitor HSP990 also suppressed BON1 cell viability (Fig. 2A, right panel). At 24 h, significant effects were observed at the highest HSP990 dose tested (100 nM; reduction of metabolic activity to ∼75% compared to controls, p<0.05). At 72 h, significant effects were observed at a HSP990 concentration as low as 5 nM (reduction of metabolic activity to ∼71%, p<0.05) with a maximum effect at 100 nM (reduction of metabolic activity to ∼21%, p<0.05). At 144 h, 5 nM HSP990 suppressed BON1 cell metabolic activity to ∼66% (p<0.05), 10 nM to ∼52% (p<0.05), 50 nM to ∼7% (p<0.05) and 100 nM to ∼3% (p<0.05). For all concentrations and time points the decrease of metabolic activity (Fig. 2A, upper panels) correlated with the decrease of DNA content (Fig. 2A, lower panels).

Treatment of human bronchopulmonary neuroendocrine NCI-H727 tumor cells with AUY922 also suppressed cell viability in a dose-dependent manner (Fig. 2B, left panel). Significant effects were observed at all times points, beginning at a treatment concentration of 5 nM and peaking at the highest concentration tested. Similar to the observed effects of AUY922, treatment with HSP990 dose-dependently suppressed NCI-H727 cell viability (Fig. 2B, right panel). Significant effects were observed at 24, 72 and 144 h with HSP990 concentrations as low as 10 nM.

Due to their slow growth rate, cell proliferation experiments with human midgut carcinoid GOT1 cells were performed for 72 and 144 h. Treatment with AUY922 dose-dependently suppressed GOT1 cell viability. Significant effects at both time points were observed with AUY922 concentrations as low as 5 nM and peaked at the highest dose tested (100 nM; Fig. 2C, left panel). HSP990 also suppressed GOT1 cell viability with a similar potency (Fig. 2C, right panel).

Table I summarizes the IC_50_ inhibitory values of AUY922 and HSP990 on proliferation of BON1, NCI-H727 and GOT1 cells (based on metabolic activity data determined by Cell Titer 96 aqueous One Solution Proliferation assay). Lowest IC_50_ values were observed for AUY922-mediated BON1 and HSP990-mediated GOT1 metabolic activity (at 144 h).

### Effect of HSP90 inhibition on cell cycle distribution of neuroendocrine tumor cells

**Figure 3.**
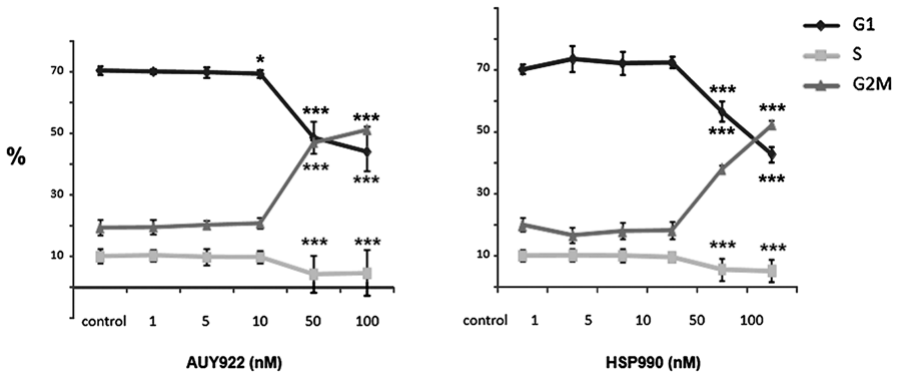
Effect of HSP90 inhibition on cell cycle distribution of neuroendocrine tumor cells. Human pancreatic neuroendocrine BON1 cells cultured in complete medium were treated with the indicated concentrations (1–100 nM) of the HSP90 inhibitors AUY922 (left panel) or HSP990 (right panel) for 24 h. Demonstrated are the mean values ± SD of 3 independently performed experiments in duplicates (n=6); p<0.001 versus untreated control.

To further explore mechanisms for the observed inhibition of neuroendocrine tumor cell viability by HSP90 inhibition, we performed cell cycle analysis of BON1, NCI-H727 and GOT1 cells treated for 24 h with AUY922 and HSP990, respectively. BON1 cells responded to AUY922 treatment with a significant reduction of cells in S phase from ∼10 to ∼5%, while the number of cells in G2/M phase was dose-dependently increased (at 50 and 100 nM; p<0.001) (Fig. 3, left panel). HSP990 also decreased the number of BON1 cells in S phase and increased the number of cells in G2/M phase with a similar potency (Fig. 3, right panel). In contrast, cell cycle phase distribution of NCI-H727 and GOT1 cells was not altered by overnight HSP inhibition (data not shown).

### HSP90 inhibition induces apoptosis in neuroendocrine tumor cells

**Figure 4.**
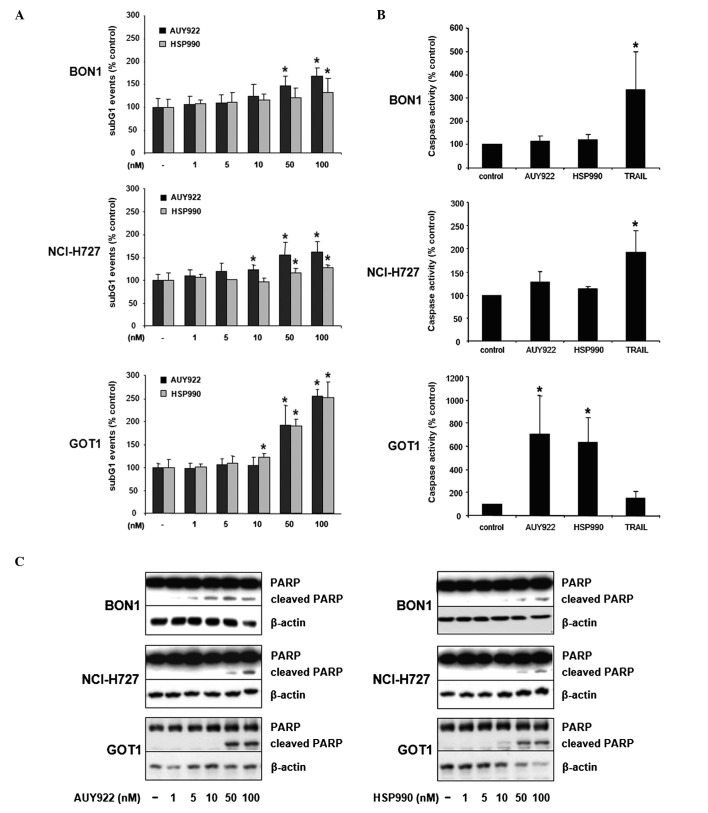
HSP90 inhibition induces apoptosis in neuroendocrine tumor cells. (A and B) BON1, NCI-H727 and GOT1 cells were treated with increasing concentrations (1–100 nM) of AUY922 or HSP990. After 24 h the proportion of cells in subG1 phase was examined by flow cytometry (A; duplicates, n=6). Additionally, cells were treated with 100 nM of AUY922 or HSP990 and TRAIL (10 ng/ml) as a positive control. After 24 h the activity of effector caspases 3 and 7 was measured with Caspase-Glo 3/7 assay (B; singular values, n=3). Shown are the mean values ± SD of three independently performed experiments. ^*^p<0.05 versus untreated control. (C) BON1, NCI-H727 and GOT1 cells were treated with increasing concentrations (1–100 nM) of AUY922 or HSP990. (C) After 24 h the extent of PARP cleavage was determined by western blot analysis.

Twenty-four hour treatment of BON1 cells with AUY922 dose-dependently increased the number of cells in sub-G1 phase up to ∼1.7-fold (100 nM, p<0.05; Fig. 4A). HSP990 also increased the number of sub-G1 events up to ∼1.4-fold (100 nM, p<0.05; Fig. 4A). Furthermore, AUY922 and HSP990 treatment resulted in a significant increase of NCI-H727 cells in sub-G1 phase up to ∼1.6-fold (100 nM AUY922, p<0.05; Fig. 4A) and ∼1.3-fold (100 nM HSP990, p<0.05; Fig. 4A), respectively. GOT1 cells showed the strongest increase of DNA fragmentation in response to HSP90 inhibition (up to ∼2.5-fold at 100 nM AUY922 or HSP990, p<0.05; Fig. 4A).

To further specify the observed HSP90 inhibition-mediated increase of the sub-G1 fraction, cells were additionally assayed for the activity of effector caspases 3 and 7. While inducing only slight increases of caspase 3/7 activity in BON1 and NCI-H727 cells, both HSP90 inhibitors induced a massive increase of caspase 3/7 activity in GOT1 cells up to ∼7.0-fold (100 nM AUY922, p<0.05; Fig. 4B). The induction of PARP cleavage confirmed the results obtained by measurement of caspase 3/7 activity, demonstrating more potent induction of PARP cleavage in GOT1 compared to BON1 and NCI-H727 cells (Fig. 4C).

### Mechanisms for HSP90 inhibition in neuroendocrine tumor cells: effects on downstream signaling

**Figure 5.**
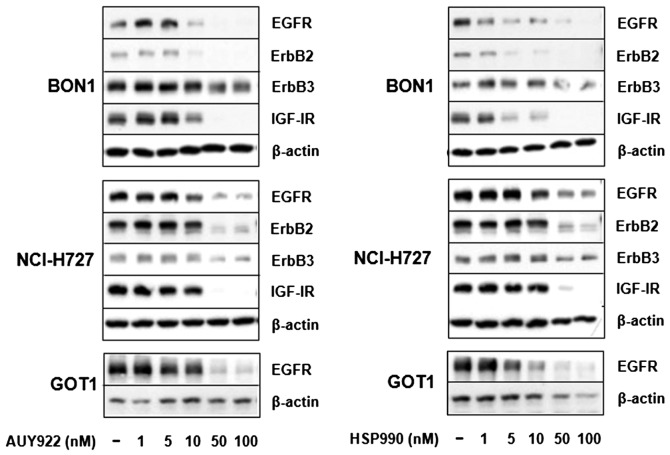
Effect of HSP90 inhibition on RTK expression in neuroendocrine BON1 tumor cells. BON1, NCI-H727 and GOT1 cells were treated with increasing concentrations (1–100 nM) of the HSP90 inhibitor AUY922 (left panel) or HSP990 (right panel) for 24 h. Subsequently the expression of EGFR, ErbB2, ErbB3 and IGFIR was evaluated by western blot analysis. A representative blot out of 3 independently performed experiments is shown.

As the HSP90 inhibitor 17-AAG has recently been reported to reduce EGFR and IGF-IR expression in the bronchopulmonary typical carcinoid cell line NCI-H727 (13,15), we examined the effect of AUY922 and HSP990 on ErbB and IGF-I receptor expression. Treatment of BON1 cells with AUY922 and HSP990 for 24 h suppressed both ErbB2 and EGF receptor expression starting at concentrations of 5 to 10 nM with minor inhibitory effects observed on ErbB3 expression (Fig. 5). In addition, strong inhibitory effects were observed on IGF-I receptor expression (Fig. 5). In NCI-H727 cells, HSP90 inhibition abolished ErbB2 and IGF-I receptor expression, while inhibitory effects were observed on ErbB3 and EGF receptor expression (Fig. 5). In contrast to BON1 and NCI-H727 cells, ErbB2, ErbB3 and IGF-I receptor are not detectable in GOT1 cells. However, treatment of GOT1 cells with AUY922 and HSP990 for 24 h strongly suppressed EGF receptor expression (Fig. 5).

**Figure 6.**
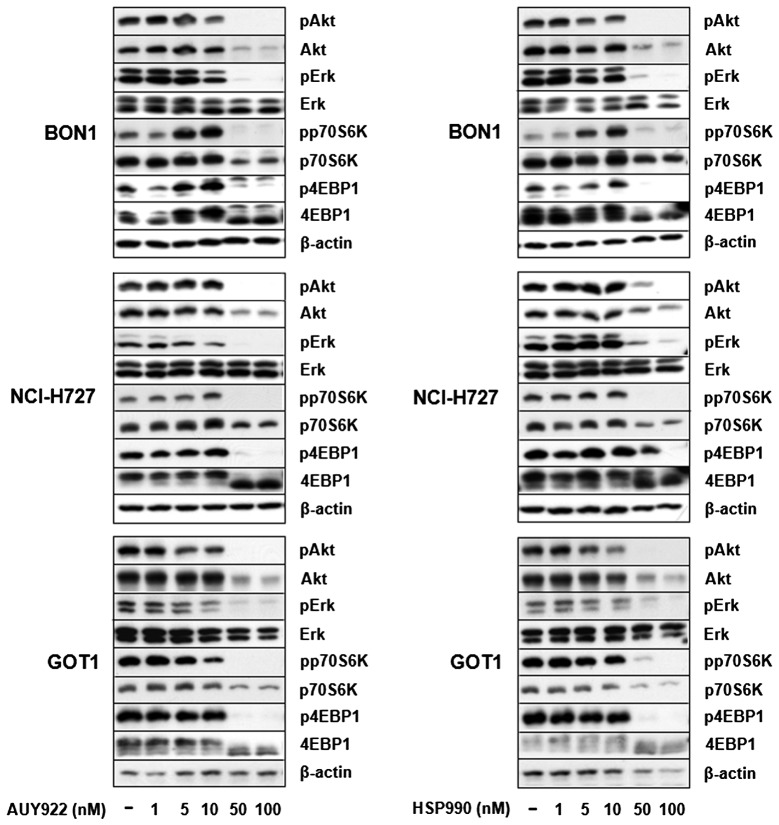
Effect of HSP90 inhibition on downstream signaling. BON1, NCI-H727 and GOT1 cells were treated with increasing concentrations (1–100 nM) of the HSP90 inhibitor AUY922 (left panel) or HSP990 (right panel) for 24 h. Subsequently, the expression of pErk1/2, Erk1/2, pAkt, Akt, pp70S6K, p70S6K, p4EBP1 and 4EBP1 was evaluated by western blot analysis. A representative blot out of 3 independently performed experiments is shown.

Untreated BON1, NCI-H727 and GOT1 cells cultured in complete medium, exhibited baseline activation of Akt and Erk signaling pathways (Fig. 6). Treatment of all cells with AUY922 and HSP990 dose-dependently suppressed Erk, Akt, p70S6K and 4EBP1 phosphorylation (Fig. 6). Potent inhibition on PI3K/Akt signaling was also demonstrated by suppression of Akt and p70S6K protein expression. Total Erk and β-actin protein expression remained unaffected at all concentrations tested (Fig. 6).

## Discussion

Due to increased incidence and relatively poor prognosis of GEP-NETs alternative treatment options are required for this heterogeneous group of tumors (5,6,9). High Akt and Erk activity in NETs as well as compensatory Akt activation in response to mTOR and Raf inhibitors, suggest that simultaneous blockade of multiple oncogenic neuroendocrine signaling cascades could be a more effective therapeutic approach (10,11). As HSP90 is overexpressed in NETs and controls the function of multiple oncogenic proteins (2,13), we examined the effect of HSP90 inhibition on neuroendocrine cell proliferation and signaling.

HSP90 inhibition was performed with novel low molecular weight ATP-competitive non-geldamycin HSP90 inhibitors AUY922 and the novel oral inhibitor HSP990. These compounds have been speculated to offer advantages over ansamycin benzoquinone HSP90 inhibitors such as 17-allylamino-17-demethoxygeldanamycin (17-AAG) based on the independence from NAD(P)H:quinone oxidoreductase 1 (NQO1) metabolism, P-glycoprotein expression and favorable aqueous solubility (16,17). All three compounds are currently in clinical trials in different solid tumor entities.

Treatment of human pancreatic BON1, bronchopulmonary NCI-H727 and midgut GOT1 carcinoid cells with increasing concentrations of AUY922 and HSP990 dose-dependently decreased cell viability. Recently, also the HSP90 inhibitors 17-AAG and IPI504 have been reported to inhibit cell viability in NCI-H727 cells with an IC_50_ value of 70.4 and 192 nM, respectively (12,13). In the current study, significant inhibition of cell viability was observed with inhibitor concentrations as low as 5 nM particularly after prolonged treatment (72–144 h). Inhibition of neuroendocrine cell viability was associated with the induction of apoptosis (especially in GOT1 cells) as demonstrated by increased number of sub-G1 events, as well as the induction of caspase 3/7 and PARP cleavage. Consistently, AUY922 has recently been demonstrated to induce apoptosis in multiple myeloma and glioblastoma cell lines (18,19).

In addition to apoptosis induction, HSP90 inhibition in human pancreatic BON1 cells was also associated with a pronounced increase of cells in G2/M phase, while no effect on cell cycle distribution was observed in NCI-H727 and GOT1 cells. Induction of apoptosis combined with inhibition of cell cycle progression (arrest in G2M phase) could explain the more potent inhibition of cell viability in BON1 compared to NCI-H727 cells. However, different growth rates of the cell lines tested may also have an impact on cell cycle analysis of non-synchronized cells at a given time point (overnight treatment).

In addition to HSP90, members of the ErbB receptor family EGFR and ErbB2, as well as IGF-IR are also expressed in NETs and could present alternative treatment targets (13,20). Here we show AUY922 and HSP90-mediated inhibition of ErbB receptor (EGFR, ErbB2 and ErbB3), and IGF-I receptor expression in BON1 and NCI-H727 cells, likely as a result of dissociation from HSP90 and subsequent increase in ubiquitinylation and proteosomal degradation (1). NETs are characterized by high activity of Erk and Akt signalling (10). Consistently, we detected baseline activity of Erk and Akt signalling pathways (phosphorylation of Erk, Akt, p70S6K, 4EBP1) in all NET cell lines tested. Neuroendocrine HSP90 inhibition suppressed both signalling pathways, possibly at least in part through depletion of upstream signalling RTKs. Interestingly, HSP90 inhibitors at lower concentrations (5–10 nM) increased p70S6K and 4EBP1 signalling in BON1 cells possibly due to incomplete HSP90 inhibition at this concentration and compensatory activation of alternate stimulatory feedback. HSP90 inhibition was not a toxic effect, since no effect was observed on Erk, or β-actin expression. Furthermore, HSP90 inhibition was associated with HSP70 upregulation, a hallmark of HSP90 inhibition.

Considering the complexity of neuroendocrine tumors, targeting a single pathway by inhibiting the activity of one component is unlikely to be effective in the long term due to development of resistance and activation of compensatory mechanisms (11). We report simultaneous suppression of neuroendocrine Erk and Akt signalling associated with induction of apoptosis and inhibition of cell viability by the novel HSP90 inhibitors AUY922 and HSP990. As HSP90 overexpression was recently reported in NETs (13) inhibition of HSP90 alone or in combination with other molecular targets could be useful for the treatment of aggressive neuroendocrine tumors resistant to conventional therapy. Further preclinical and clinical studies are needed to evaluate the potential role of HSP90 inhibitors in neuroendocrine tumors.

## Figures and Tables

**Table I.** IC_50_ values (nM) of AUY922 and HSP990-mediated inhibition of NET cell proliferation at 72 and 144 h.

	AUY922		HSP990	
	72 h	144 h	72 h	144 h
BON1	4.4	4.4	22.1	11.8
NCI-H727	12	4.6	50.9	19
GOT1	30.3	5.1	26.2	9.4
